# Effect of health education on severe thalassemia prevention and control in communities in Cambodia

**DOI:** 10.1186/s13690-018-0259-3

**Published:** 2018-02-19

**Authors:** Kimhaung Cheng, Supan Fucharoen, Kanokwan Sanchaisuriya, Goonnapa Fucharoen, Pattara Sanchaisuriya, Arunee Jetsrisuparb

**Affiliations:** 1 Graduate School, Khon Kaen University, Khon Kaen, 40000 Thailand; 2 Centre for Research and Development of Medical Diagnostic Laboratories (CMDL), Faculty of Associated Medical Sciences, Khon Kaen University, Khon Kaen, 40000 Thailand; 3 Department of Nutrition, Faculty of Public Health, Khon Kaen University, Khon Kaen, 40000 Thailand; 4 Department of Pediatrics, Faculty of Medicine, Khon Kaen University, Khon Kaen, 40000 Thailand

**Keywords:** Cambodia, Health education, Prevention and control program, Screening test, Thalassemia

## Abstract

**Background:**

Severe thalassemia diseases are a major health problem in Southeast Asia. In Cambodia, there has never been a significant program for prevention or control of severe thalassemia. We, therefore, studied the effect of a health education program on severe thalassemia prevention and control in Phnom Penh, Cambodia.

**Methods:**

A quasi-experimental study in several communities around Phnom Penh was done. The respective intervention and control group comprised 124 and 117 people, between 18 and 40 years of age, male and female. Pre- and post-tests using a validated and reliable questionnaire were performed in the intervention group and one test was done in the control group. A health education program was organized to give important information to the intervention group and, at the end of the process, to the control group. The outcomes were evaluations of their knowledge and attitude vis-à-vis severe thalassemia prevention and control, and participating in thalassemia screening.

**Results:**

Among participants in the intervention group, 105 (84.7%) considered undergoing blood screening vs. 65 (55.6%) in the control group (*p*-value < 0.001). In the intervention group, the respective mean scores for knowledge and attitude to a prevention and control program for severe thalassemia before and after health education were 2.6 VS 6.5 (*p*-value < 0.001) and 4.6 VS 6.5 (*p*-value < 0.001).

**Conclusions:**

The intention to undergo screening was significantly higher in the intervention group than the control group. Knowledge and attitude towards prevention and control of severe thalassemia was significantly improved in the intervention group. Health education clearly heightens awareness and improves consideration of screening for prevention and control of severe thalassemia.

## Background

Thalassemia is a congenital disorder of blood components caused by the absence, or decrease, of one or more of the globin chains in red blood cells, and it represents a serious global public health concern [1]. Over 56,000 affected babies are born annually and at least 5.2% of the global population are carriers. The tropics have the highest incidence, where 7% of pregnant women are carriers [2, 3].

Thalassemia can be classified by clinical manifestations as thalassemia major, intermedia, or minor. Thalassemia major comprises patients with a hemoglobin level < 7.0 g/dL, requiring regular blood transfusions to survive and life-long use of iron chelation therapy for treating iron overload. Thalassemia major includes homozygous β^0^-thalassemia, some cases of Hb Eβ-thalassemia, and homozygous α^0^-thalassemia 1 (Hb Bart’s hydrops fetalis syndrome) [4]; the latter being the most severe form from which patients usually die in utero or soon after birth [5]. Patients with thalassemia intermedia (also known as non-transfusion dependent thalassemia – NTDT) have clinical manifestations, presenting somewhere between thalassemia major and minor. The symptoms are mild to moderate anemia, with a Hb level ≥ 7.0 g/dL, and the patient occasionally needs blood transfusions [6].

As severe thalassemia affects both physical and mental health, it causes patients and their parents suffering; therefore, prevention and control of severe thalassemia are important. At present, several countries have comprehensive national prevention programs for severe thalassemia—including Italy, Greece, Cyprus, the UK, France, Iran, Thailand, Australia, Singapore, Taiwan, Hong Kong, and Cuba [7]. The process includes public awareness and health education, carrier screening, genetic counseling, prenatal diagnosis, and sometimes therapeutic abortion when a life-threateningly severe form of thalassemia is confirmed. Limited programs—including antepartum screening according to ethnic group—are available in only a few countries in northern Europe [8]. The common types of thalassemia in Southeast Asia are α^0^-thalassemia, α^+^-thalassemia, β^0^-thalassemia, hemoglobin (Hb)E, and Hb Constant Spring [9]. In Cambodia, the respective reported prevalence of α-thalassemia, β-thalassemia, and Hb E is 30–40%, 0.8–1.1%, and 13.9–33.1% [10].

The Pol Pot Khmer Rouge regime in Cambodia resulted in population displacement, catastrophic human suffering and death, and political conflict [11]. After the regime fell, health care was one of the most difficult services to restore as the country lacked educated manpower and professionals (i.e., doctors, nurses, pharmacists). Even today, the majority of Cambodians do not attend high school or higher education [12]. Cambodia is now undergoing restoration of its economic and social development and the Cambodian Ministry of Health is endeavoring to improve its mission and working principles for addressing severe thalassemia.

### Objective

The objective of this study was to evaluate the effects of health education on severe thalassemia prevention and control program in communities around Phnom Penh, Cambodia. Our hypothesis was that after implementing health education on thalassemia, the intervention group would have more knowledge and an improved attitude towards severe thalassemia prevention and control, and would have greater participation in screening for severe thalassemia than the control group.

## Methods

This was a quasi-experimental study—with an intervention and control group—conducted in 4 communities around Phnom Penh, Cambodia. The distance between each community was around 10 km—a 30 min drive over barely passable roads, so it was unlikely participants in different villages would exchange information; thereby providing a putative mechanism for preventing cross-contamination. There has, moreover, never been a significant program for prevention or control of severe thalassemia in Cambodia, so we assumed cross-contamination between groups would be minimal.

### Setting

We had 4 communities prepared and KC gave the inclusion and exclusion criteria to each village head. After going to the first 3 villages, we found that the control group had markedly fewer participants simply because fewer people came than we expected than the intervention group. We, therefore, added another community, “Orn Long Kong”. We then divided the participants in this community into 2 groups so as to balance the intervention and control groups. It was only in this community that we color coded and separated participants for prevention of informational cross-contamination: this method was not used in the other communities.

In the end, among the 4 communities, we randomly selected the control and intervention groups. Khva Choung Toul community, Khva Choung Toul village, Dorng Khou commune, Dorng Khou district, Phnom Penh, and Toul Sambo community, Toul Sambo village, Prey Veng commune, Dorng Khou district, Phnom Penh constituted the control group; while Khva Kdey Songkhom community, Khva Choung Toul village, Dorng Khou commune, Dorng Khou district, Phnom Penh constituted the intervention group and Orn Long Kong community, Toul Sambo village, Prey Sor commune, Dorng Khou district, Phnom Penh, constituted both control and intervention groups. Participants were invited to join the study by the village head according to inclusion criteria. The study was conducted between June 2016 and December 2016. The field study was run over three consecutive days to avoid any potential cross-contamination between groups.

For the control group, a questionnaire was given to assess their knowledge and attitude regarding thalassemia. In the control group, the questionnaire was marked black with a running research number for each. After collecting the questionnaire, a screening test was offered free of charge. Health education was thus provided to them and a post-test was not given to the control group.

For the intervention group, the pre-test questionnaire was marked with a running research number for each respondent. After collecting the pre-test, the health education program was given to the participants; after which the post-test was given—using the same questionnaire with a running research number for each respondent. (The socio-demographic part was not given twice.) In the intervention groups, we gave the second questionnaire just after the health education finished. The rationale for this interval was that their acquired knowledge of thalassemia and attitude towards prevention and control of severe thalassemia were the result of the intervention that we provided and was not due to any contamination from other means including more time to study.

After finishing the post-test, blood screening was also offered to all participants. For the intervention group in Orn Long Kong community, the pre-test questionnaire was marked red and the post-test blue; both with a running research number for each respondent. The control and the intervention groups were separated to prevent cross-contamination or peer influences.

Individuals were interviewed using a questionnaire to gather information about their knowledge and attitude toward severe thalassemia prevention and control. The questionnaire developed by Jopang et al. [13] for participants between 18 and 40 was translated into Khmer by a certified organization and validated by 1 doctor and 3 nurses. A reliability test for the attitude towards severe thalassemia prevention and control—which had only 2 answers (agree or disagree)—was performed on 26 participants before using it as the tool (*P* = 0.76 by Kuder and Richardson Formula 21) [14]. The 26 participants (i.e., 4 less than we expected) lived in Samaki community in Toul Kork Khan, Phnom Penh, Cambodia and were not participants in the main study. The interval between the reliability test and the main study was around 2 months.

### Participants

The inclusion criteria were (a) people of reproductive age between 18 and 40; (b) either male or female; and, (c) able to read and write. The study included healthy subjects, but excluded vulnerable subjects. The exclusion criteria were (a) people with a mental defect; (b) pregnant women; and, (c) women with their last menstrual period (LMP) more than 6 weeks prior. Pregnant women were excluded because in Cambodia, there is no system for prevention and control of severe thalassemia, so if a woman discovered that she had an affected fetus it could cause anxiety. Similarly, in Thailand, women 24 weeks pregnant are not screened because it is too late for a therapeutic abortion.

### Variables and measurement

Knowledge about thalassemia and attitude towards the prevention and control of severe thalassemia were assessed through use of a questionnaire: (a) before and after health education in the intervention group; and, (b) once in the control group. The questionnaire (Supplementary data) comprised 3 parts: Part 1 to assess the socio-demographic background of participants; Part 2 to assess knowledge of thalassemia through multiple choice answers (8 items on genetic background, prevention, and treatment of the disease); and, Part 3 to assess attitude (8 items on blood screening, prenatal diagnosis, and consequences of abortion). Evaluations of both knowledge and attitude towards the prevention and control of severe thalassemia and the number of participants who decided to have blood test were the outcomes.

The questions related to knowledge and attitude about thalassemia were scored (i.e., 0 for an incorrect response, and 1 for a correct response). A well-organized training program was conducted to give basic information and the key points to the villagers; focusing on the nature of the disease and how patients/carriers pass on the disease to their offspring. In order to avoid passing on severe thalassemia, an individual of reproductive age who plans on having children should undergo screening before pregnancy (or at the early stages thereof). The safety, accuracy, and cost of screening were taught through use of posters and flipcharts.

Screening was done on-site using the osmotic fragility (OF) test [15]. The program was run with participation of laboratory staff from the National Pediatric Hospital, Cambodia, and with the help of staff from the Faculty of Associated Medical Sciences and Centre for Research and Development of Medical Diagnostic Laboratories, Khon Kaen University, Thailand (CDML). The remaining blood samples were kept at the recommended temperature and safely transferred to the CMDL for further Hb-typing and DNA analysis by PCR.

### Bias

We randomly assigned communities into the intervention and control groups. The communities had similar conditions and the participants similar between groups.

### Study sample size calculation

The sample size was calculated using a formula adapted from the dissertation by Jopang et al. [13]. Thalassemia was studied using the Health Belief Model in a high school; knowledge and attitudes of the participants was assessed according to outcomes described by Lagampan et al. [16] and Jopang et al. [13]. The study showed that a respective 73.7% and 57.7% of students in the intervention and control group were willing to do blood screening test [16].

### Statistical methods

STATA version 13 was used for data entry (double-entry) and analysis. The socio-demographic characteristics of the participants were analyzed using descriptive statistics (i.e., frequencies, means, standard deviation, median, and range). The relationship between variables and outcomes were analyzed using univariate and multivariate logistic regression. A *p*-value of less than 0.05 was considered statistically significant.

## Results

Seventy-one villagers did not meet the inclusion criteria because of (a) illiteracy, (b) age (over 40 or under 18), or (c) pregnancy. In the final analysis, 241 villagers were recruited (124 and 117 in the intervention and control groups, respectively). Univariate analysis demonstrated that only the age of participants was significantly different between the intervention and control groups (Tables 1 & 2). Most participants had never heard of thalassemia. More than 50% of our participants had only completed primary school and the majority were factory workers.

**Table 1:** Socio-demographic characteristics of the participants

	Intervention group (*N* = 124)	Control group (*N* = 117)	*P*-value
Sex			0.06
Male	12 (9.7)	21 (17.9)	
Female	112 (90.3)	96 (82.1)	
Age (18–40 years old)			0.04
< 25	37 (29.8)	28 (23.9)	
25–30	44 (35.5)	17 (14.5)	
31–40	43 (34.5)	72 (61.5)	
Mean (SD)	28.5 (6.3)	30.2 (6.8)	
Median (range)	28 (18 to 40)	32 (18 to 40)	
Education			0.29
Primary school	64 (51.6)	64 (54.7)	
Secondary school	31 (25.0)	33 (28.2)	
High school	17 (13.7)	15 (12.8)	
Bachelor degree	12 (9.7)	5 (4.3)	
Main occupation			0.10
Factory worker	63 (50.8)	47 (40.2)	
Unemployed	24 (19.4)	27 (23.1)	
Own business	13 (10.5)	22 (18.8)	
Agriculture & labor	12 (9.7)	15 (12.8)	
Teacher	12 (9.7)	6 (5.1)	
Household (Reil/month)* *4000 Riels = 1USD			0.23
< =500,000	57 (46.0)	46 (39.3)	
500,100–990,000	33 (26.6)	50 (42.7)	
> =1,000,000	34 (27.4)	21 (18.0)	
Mean (SD)	670,967 (297,076.3)	609,658.1 (264,327.2)	
Median (range)	600,000 (120,000 to 2,000,000)	600,000 (140,000 to 2,000,000)	
Household (Reil/month)* *4000 Riels = 1USD			0.23
< =500,000	57 (46.0)	57 (46.0)	
500,100–990,000	33 (26.6)	33 (26.6)	
> =1,000,000	34 (27.4)	34 (27.4)	
Mean (SD)	670,967 (297,076.3)	670,967 (297,076.3)	
Median (range)	600,000 (120,000 to 2,000,000)	600,000 (120,000 to 2,000,000)	
Self-social economic perception			0.71
Poor	84 (67.8)	83 (70.9)	
Average	37 (29.8)	29 (24.8)	
Above average	3 (2.4)	5 (4.3)

**Table 2:** Characteristics for medical history of individuals from the intervention and the control groups

	Intervention group (N = 124)	Control group (N = 117)	*P*-value
Frequency to see doctor			0.43
Never	43 (34.7)	33 (28.2)	
Once/year	25 (20.2)	19 (16.2)	
2–5 times/year	35 (28.2)	47 (40.2)	
6–24 times/year	21 (16.9)	18 (15.4)	
Reason to see doctor			0.67
Check up	59 (47.6)	57 (48.8)	
Follow-up (chronic diseases)	22 (17.7)	27 (23.0)	
None	43 (34.7)	33 (28.2)	
Right to health coverage			0.60
Self-pay	109 (87.9)	99 (84.6)	
Social security, welfare official & non-government organization	15 (12.1)	18 (15.4)	
Number of living child			0.39
No child	37 (29.8)	32 (27.4)	
1–2 child (children)	64 (51.6)	53 (45.3)	
> =3children	23 (18.5)	32 (27.3)	
Mean (SD)	2.16 (1.3)	2.38 (1.4)	
Median (range)	2 (1 to 7)	2 (1 to 8)	
Number of child mortality			0.05
None	116 (93.6)	106 (90.6)	
1 child	7 (5.7)	2 (1.7)	
2 children	1 (0.8)	5 (4.3)	
> =3 children	0 (0)	4 (3.4)	
Mean (SD)	1.12 (0.35)	1 (1 to 2)	
Median (range)	2.5 (1.29)	2 (1 to 5)	
Heard about thalassemia			0.07
Never	110 (88.7)	112 (95.7)	
Ever	14 (11.3)	5 (4.3)	
Means of hearing about thalassemia			0.31
None	110 (88.7)	112 (95.7)	
Health center & doctor/nurse	11 (8.9)	1 (0.9)	
Friend & family/ relatives	3 (2.4)	4 (3.4)	
Having thalassemia child			0.30
None	124 (100)	116 (99.2)	
Have	0 (0)	1 (0.8)	
Having thalassemia relative			0.96
None	123 (99.2)	116 (99.2)	
Have	1 (0.8)	1 (0.8)	

The unpaired t-test revealed that knowledge and attitude towards the prevention and control of severe thalassemia in the control and intervention groups before health education was not significantly different (Table 3). The correct knowledge and agreement attitude towards the prevention and control of severe thalassemia were significantly increased in all items after health education in the intervention group (Tables 4 & 5). The respective mean scores in the intervention group before and after health education were 2.6 VS 6.5 and 4.6 VS 6.5 for knowledge (*p*-value < 0.001) and attitude towards the prevention and control of severe thalassemia (*p*-value < 0.001) (Table 6).

**Table 3:** Comparison of thalassemia knowledge and attitude scores of individuals from the intervention and the control groups before conducting the health education

Thalassemia knowledge and attitude	Intervention group (*N* = 124)	Control group (*N* = 117)	*P*-value
Knowledge of thalassemia score			0.09^a^
Mean (SD)	2.6 (1.1)	2.9 (1.3)	
Median (range)	3 (0 to 5)	3 (0 to 7)	
Attitude towards thalassemia score			0.20^a^
Mean (SD)	4.6 (1.8)	5.1 (1.6)	
Median (range)	5 (1 to 8)	5 (1 to 8)	

^a^ Mann-Whitney U-test

**Table 4:** Comparison of the proportion of correct knowledge before and after health education program among 124 participants in the intervention group

Thalassemia knowledge	Before education n (%)	After education n (%)	Difference (%)	95% CI
1. Congenital disease	50 (40.3)	113 (91.1)	50.8	40.0 to 61.5
2. Knowing carrier status requiring blood test	83 (66.9)	121 (97.6)	30.7	21.5 to 39.7
3. Thalassemia carrier is a healthy person but carrying thalassemia gene	22 (17.7)	89 (71.8)	54.1	44.3 to 63.7
4. The affected children resulted from parents who might pass the disease to their children	53 (42.7)	108 (87.1)	44.4	34.1 to 54.5
5. Chance of having thalassemia child	18 (14.5)	115 (92.7)	78.2	70.8 to 85.5
6. Pre-conception diagnosis for both parents is the best way to prevent thalassemia kid/s	66 (53.2)	114 (91.9)	38.7	29.7 to 47.6
7. Prevention of the disease	13 (10.5)	88 (71.0)	60.5	50.9 to 70.0
8. Treatment of the disease	11 (8.9)	90 (72.6)	63.7	54.2 to 73.1

**Table 5:** Comparison of the proportion of agreement attitude before and after health education program among 124 participants in the intervention group

Thalassemia attitude	Before education n (%)	After education n (%)	Difference (%)	95%CI
1. Willing to join in thalassemia screening	51 (41.1)	90 (72.6)	31.5	20.0 to 42.8
2. I will have a blood test even no relative is known to be a patient suffering from thalassemia.	70 (56.5)	111 (89.5)	33.0	22.0 to 43.8
3. My partner is important to do blood test if I carry a mutated thalassemia gene	39 (31.5)	58 (46.8)	15.3	3.0 to 27.5
4. Willing to take partner to consult MD during pregnancy if both carry a mutated thalassemia gene	79 (63.7)	110 (88.7)	25.0	14.0 to 35.5
5. Willing to consult physician for PND of thalassemia	63 (50.8)	108 (87.1)	36.3	26.0 to 46.5
6. Willing to operate for abortion in case fetus with severe thalassemia disease	67 (54.0)	116 (93.6)	39.6	30.0 to 48.8
7. Willing to consult MD for family planning if I have a thalassemia child	95 (76.6)	121 (97.5)	20.9	12.0 to 29.2
8. Willing to do blood screening to prevent baby suffering from severe thalassemia	71 (57.2)	93 (75.0)	17.8	5.0 to 29.9

**Table 6:** Mean (SD) of knowledge and attitude scores before and after health education among 124 participants

	Before education	After education	*P*-value
1. Thalassemia knowledge	2.6 (1.1)	6.5 (1.1)	< 0.001
2. Thalassemia attitude	4.6 (1.8)	6.5 (1.3)	< 0.001

The proportion of participants willing to have a blood test in the intervention group was significantly higher than in the control group. Among the 124 participants in the intervention group, 105 (84.7%) agreed to do blood screening compared to 65 (55.6%) in the control group (*p*-value < 0.001) with an odds ratio (OR) of 4.3 (95% CI, 2.4 to 7.9). (Table 7). The crude odds ratio of all factors that might affect participation in the blood test in the intervention group was calculated using a univariate analysis (Table 8). The factors with a *p*-value < 0.25 were selected for use in the multivariate analysis (Table 9). Among those who had blood tests (170 participants), 120 (70.6%) were positive for the OF test: 1 (0.6%) had α^0^- thalassemia (SEA deletion); 2 (1.2%) were Hb E homozygous; 50 (29.4%) were Hb E heterozygous; and, 0 had β-thalassemia (Table 10).

**Table 7:** The effect of health education in consideration to have a blood test between the intervention group and the control group

Factors	Number of participants who had a blood test	(%)	OR	95% CI	*P*-value
Group					0.001
Control group (n = 117)	65	55.6	1		
Intervention group (n = 124)	105	84.7	4.3	2.4 to 7.9

**Table 8:** Crude OR of all factors that might affect participation in blood testing (*n* = 105) in the intervention group. *P*-value < 0.25 is being selected

Factors	Number of participants who had a blood test	(%)	Crude OR	95% CI	*P*-value
1. Sex					0.09
Male	8	66.7	1		
Female	97	86.6	3.2	0.9 to 12.1	
2. Congenital disease					0.001
No	5	45.4	1		
Yes	100	88.5	9.2	2.5 to 34.5	
3. Prevention of the disease					0.18
No	28	77.8	1		
Yes	77	87.5	2	0.7 to 5.5	
4. Willingness to join in thalassemia screening					0.001
Disagree	17	50.0	1		
Agree	88	97.8	44	9.3 to 208.2	
5. I will take my partner for screening test if I carry a thalassemia gene					0.01
Disagree	51	77.3	1		
Agree	54	93.1	4	1.2 to 12.7	
6. Attitude toward termination of pregnancy of a severe thalassemia fetus					0.11
Disagree	5	62.5	1		
Agree	100	86.2	3.7	0.8 to 17.2	
7. Perception on preventing severe thalassemia baby					0.21
Disagree (*n* = 31)	24	77.4	1		
Agree (*n* = 93)	81	87.1	1.9	0.7 to 5.5

**Table 9:** Factors affecting participation in blood testing in the intervention group. *P*-value < 0.05 is considered as a significant factor

Factor	Number	Blood test (%)	Adjust OR	95% CI of adj.OR	*P*-value
1. Congenital disease					0.03
No	5	45.5	1		
Yes	100	88.5	20.6	1.3 to 323	
2. Willingness to join thalassemia screening					0.001
Disagree	17	50.0	1		
Agree	88	97.8	185	16.1 to 2122	
3. Attitude toward termination of pregnancy of a severe thalassemia fetus					0.045
Disagree	5	62.5	1		
Agree	100	86.2	19	1.1 to 338.5	
4. Perception on preventing severe thalassemia baby					0.035
Disagree	24	77.4	1		
Agree	81	87.1	7.4	1.1 to 47.9

**Table 10:** Three categories of thalassemia found among 170 individuals who participated in blood screening

Type of thalassemia	Number	Percent (%)
Hb E heterozygous	50	29.4
Hb E homozygous	2	1.2
α^0^ - thalassemia (SEA deletion) carrier	1	0.6

## Discussion

Even though there is a reported high prevalence of thalassemia in Cambodia [10], we did not encounter many cases of severe thalassemia carriers (α^0^- and β^0^-thalassemia) in the current community-based study. This study’s result was similar to a recent community-based study by Karakochuk et al. [17]. We documented a low level of knowledge about thalassemia in these communities and highlighted the urgency for implementation of effective health education. The respective mean score of knowledge and attitude vis-à-vis the prevention and control of severe thalassemia in the intervention group was significantly increased after health education on thalassemia, and as hypothesized consideration to undergo thalassemia screening was significantly increased in the intervention group.

Severe thalassemia patients experience serious physical and psychological sequelae and need proper coping strategies and lifelong support from family and health services. Prevention and control of severe thalassemia is more effective than treatment [18, 19]. Severe thalassemia diseases can be prevented and health education is the most powerful first step for increasing knowledge and awareness [8]; the goal being an informed choice among carriers about getting a screening test. Most participants in the current study—whether in the intervention or control group were female (90.3% and 82.1%); similar to studies in Italy, Iran, and Thailand, where there are successful national prevention and control policies for severe thalassemia [20].

Zaman and Salahuddin (2006) found an association between the level of education and the level of awareness of thalassemia, such that people with higher education have greater appreciation of the risk of thalassemia [21]. In fact, awareness campaigns vis-à-vis thalassemia have been shown to be effective. The high level of awareness among Italians is due to aggressive public health measures, including thalassemia-carrier screening and genetic counseling [22]. The health care providers in Cyprus, Greece, Iran, and Italy have used national premarital screening for thalassemia as a standard practice, which helps in successful identification of at-risk couples needing genetic counseling [22]. Similarly, although most of the participants in our intervention group (51.6%) had only primary school and 25% had secondary school, their knowledge of thalassemia and attitude towards prevention and control of severe thalassemia were both significantly increased after health education on thalassemia.

The present study’s participants were between 18 and 40 years of age. Since 18-year-olds normally have a high school education and 40-year-olds are more likely to have a higher academic degree, these education realities we thought would have an impact on knowledge and attitude. In fact, the level of education in the present study was not a significant factor perhaps because of the small sample size or the older participants had never had opportunity to study due to the political conflict in Cambodia [11].

By way of corroboration, a significantly higher number of participants underwent a blood check in the intervention group (84.7%) compared to 56% in the control group (*p*-value < 0.001). Therefore, health education proved to be a powerful tool in this pilot prevention and control program for severe thalassemia in Cambodia. The odds ratio presented in Table 7 demonstrates that the probability of participants’ health education in the intervention group considering getting a blood test for thalassemia was 4.3 times that of the control group. Factors affecting participation in blood testing in the intervention group are presented in Table 9. Despite having a significant effect (*p*-value < 0.05), the 95% CIs were wide, reflecting our small sample size. The factors in Table 9, age and education level should, nevertheless, be considered as potentially contributing factors whenever health education in thalassemia is provided. The other factors did not result in any significant difference vis-à-vis consideration of blood testing but should not be discounted until a study with a larger sample size is conducted.

The health belief model (HBM) was substituted with its 4 structures symbolizing the perceived threat and net benefits: namely perceived susceptibility, severity, benefits, and barriers. The readiness to act depends on the concepts of people, cues to action, which would stimulate an overt behavior. A recent addition to the HBM is the concept of self-efficacy, or one’s confidence in successful action. The HBM concept was formulated to help people more effectively change unhealthy behaviors, such as being sedentariness, smoking, or overeating [23]. In an HBM study of high school students, Lagampan et al. showed that 73.7% of students in an intervention group and 57.7% in a control group were willing to undergo blood screening for severe thalassemia [16].

Our results also demonstrated that females underwent blood testing more than males. This was the experience in other studies, where females were more interested in and concerned about health prevention and health care than males [24]. In the control group, 5 female participants changed their mind about getting a blood test after health education; confirming an effect for health education. We found that one-third of the participants in both groups had never gone to see a doctor (34.7% and 28.2%, respectively), and nearly all of them (88.7% and 95.7%, respectively) had never heard about thalassemia.

In the end of the study, some participants in the intervention group did not consider getting screening because they had competing priorities and/or lacked the time to wait for the procedure, suggesting if they had the time they might get the test. It would, therefore, be cost effective to initiate laboratory facilities for the detection of α^0^- and β^0^-thalassemia among individuals and couples in Cambodia.

## Conclusions

The current study demonstrated the effectiveness of health education vis-à-vis community-based prevention and control of severe thalassemia in Phnom Penh, Cambodia. A key for success is having a competent medical team involved. A laboratory strategy for severe thalassemia prevention and control program in Cambodia might be different from other countries that also have a high prevalence of α^0^- and β^0^-thalassemia.

### Limitations

The study sample size was calculated using a formula adapted from Jopang et al. (11), which indicated that 137 participants were needed for each group. It turned out that we had unintentionally included several participants under 18 and over 40 but these were not included in the statistical analysis.

Concerning socioeconomic status, direct questions for evaluating the level of income may result in information bias. In order to evaluate this factor, an economic index and concentration index should lead to a more accurate evaluation of socioeconomic status. The economic index—a statistical measure of changes in a representative group of individual data points—may be derived from any number of sources, including company performance, prices, productivity, and employment. The concentration index is a frequently used indicator of socioeconomic inequality of health. We, however, used a less complicated means for evaluation because ours was not a large-scale study; thus, we evaluated occupation, income, and self-evaluation of their economic status as in Jopang’s study of northeastern Thailand.
